# A Graph Convolutional Network-Based Fine-Grained Low-Latency Service Slicing Algorithm for 6G Networks †

**DOI:** 10.3390/s25103139

**Published:** 2025-05-15

**Authors:** Yuan Ye, Caiming Zhang, Chenlan Wu, Xiaorong Zhu

**Affiliations:** College of Telecommunications and Information Engineering, Nanjing University of Posts and Telecommunications, Nanjing 210003, China; 1024010410@njupt.edu.cn (Y.Y.); 1223013709@njupt.edu.cn (C.Z.);

**Keywords:** 6G networks, low-latency services, network slicing, graph convolutional network

## Abstract

The future 6G (sixth-generation) mobile communication technology is required to support advanced network services capabilities such as holographic communication, autonomous driving, and the industrial internet, which demand higher data rates, lower latency, and greater reliability. Furthermore, future service classifications will become more fine-grained. To meet the requirements of these low-latency services with varying granularities, this work investigates fine-grained network slicing for low-latency services in 6G networks. A fine-grained network slicing algorithm for low-latency services in 6G based on GCNs (graph convolutional networks) is proposed. The goal is to minimize the end-to-end delay of network slicing while meeting the constraints of computational resources, communication resources, and the deployment of SFCs (service function chains). This algorithm focuses on the construction and deployment of network slices. First, due to the complexity and diversity of 6G networks, DAGs (Directed Acyclic Graphs) are used to represent network service requests. Then, based on the depth-first search algorithm, three types of SFCs of latency-type network slices are constructed according to the available computing and communication resources. Finally, the GCN-based low-latency service fine-grained network slicing algorithm is used to deploy SFCs. The simulation results show that the latency performance of the proposed algorithm outperforms that of the Double DQN and DQN algorithms across various scenarios, including changes in the number of underlying network nodes and variations in service sizes.

## 1. Introduction

The rapid progression of mobile communication technologies has heightened the necessity for the advanced exploration of network slicing architectures. Existing research predominantly concentrates on SFC mapping and deployment methodologies. Research on SFC deployment mostly focuses on the traditional chained SFC, and its structure and characteristics are discussed in depth. As outlined in [1], a node-ranking mechanism is implemented at the node mapping layer, leveraging network topology attributes and neighboring node significance. For link mapping optimization, a multi-constraint link mapping model is established, with genetic algorithms employed to identify optimal mapping paths, thereby enhancing resource allocation efficacy. In reference [2], a collaborative virtual node–link mapping algorithm is proposed to ensure the balanced utilization of the physical node and link resources in network slicing. Reference [3] introduces a radio access network slicing-based framework for user access control and wireless resource allocation, specifically tailored for smart grid applications. In [4], a novel delay-sensitive 5G access network slicing is proposed that can meet the demands of applications with different traffic characteristics, providing them with varying rate and delay guarantees to ensure efficient quality of service realization. However, with the continuous development of network technology and the increasing diversification of application requirements, the form and deployment mode of SFCs are characterized by more flexibility and complexity. In view of the complexity and diversity of current services, the traditional chained SFC is no longer able to fully describe and satisfy diverse service requests.

Concurrently, the effective utilization of network topology information has garnered increased attention. Current approaches predominantly utilize complex network theory to define node performance metrics through topological analysis. Reference [5] presents a graph-theory-driven resource scheduling algorithm incorporating depth-first search optimization, which satisfies latency, data rate, and reliability requirements for power services while reducing end-to-end delays. As outlined in [6], node importance metrics are derived from comprehensive topological evaluations of sliced and substrate networks, enabling precise node prioritization during mapping processes to optimize resource utilization.

The progressive evolution of communication technologies has exacerbated the limitations of conventional coarse-grained network slicing paradigms in addressing the escalating complexity of low-latency service requirements. This technological gap has driven substantial research focus toward fine-grained network slicing implementations. In the realization of network slicing technology, the choice of slice granularity is of high significance. References [7,8] show that if the granularity is too coarse, the flexibility of slices and the diversity and independence of user services will be limited, but if the granularity is too small, the management difficulty of different slices and the difficulty of sharing resources among slices will be increased. With the continuous development of various new services, the current demand for QoS is increasing, and in order to ensure better performance, it is necessary to move closer to a finer granularity. In reference [9], the concept of link-level network slicing is proposed, necessitating resource provisioning enforcement at the physical link stratum rather than conventional slice-level abstraction. This approach enables direct resource allocation to communication links through advanced fading mitigation and interference cancellation techniques, thereby theoretically guaranteeing QoS compliance. Nevertheless, per-service link-level slice instantiation inevitably incurs resource fragmentation, diminishing infrastructure utilization efficiency. Addressing massive IoT deployments in smart environments, reference [10] extends conventional 5G service taxonomies through micro-slice isolation mechanisms. The proposed architecture enables localized logical subnetworks between device clusters and application servers, featuring duration-aware and coverage-adaptive slice customization. Reference [11] investigates the problem of slice-based service function chain embedding (SBSFCE) to enable end-to-end network slice deployment. Simulations show that the algorithm outperforms the benchmark algorithm. Reference [12] uses machine learning to analyze network traffic and conducts experiments on different machine learning models. Based on these experimental results, a 5G slice management algorithm is proposed to optimize the slice resource. In reference [13], the author proposes an MEC network slice-aware smart queuing theoretical architecture for next-generation pre-6G networks by optimizing slice resources to improve performance metrics for both online and offline scenarios. While such micro-slices permit dynamic reconfiguration via application-layer policy orchestration with elevated automation levels, their inherent control-loop latencies render them suboptimal for stringent latency-sensitive applications. Consequently, systematic methodologies for constructing and deploying fine-grained latency-constrained network slices remain an open research challenge.

Current research paradigms in network slicing resource management predominantly address singular resource types or unilateral optimization of either RAN (radio access network) or core network components, neglecting cross-domain optimization frameworks and multi-resource interdependencies. In practical deployment scenarios, heterogeneous resource allocation across multiple slices exhibits multidimensional coupling characteristics, necessitating systematic investigation of joint multi-resource orchestration strategies integrated with end-to-end RAN–core network co-optimization. Furthermore, while existing SFC deployment studies have extensively examined conventional linear service function chains through structural and characteristic analyses, the evolution of network architectures and the diversification of application requirements have precipitated the emergence of hyper-connected service graphs. These graph-structured SFCs demonstrate enhanced topological flexibility but introduce non-trivial deployment complexity, rendering traditional chain-based models inadequate for contemporary service provisioning. Consequently, pioneering research into graph-theoretic SFC deployment mechanisms becomes imperative, particularly focusing on neighborhood-aware resource coordination algorithms and topology-adaptive scheduling paradigms to achieve precise service differentiation and infrastructure efficiency maximization.

## 2. System Model

The advent of 6G networks has been propelled by groundbreaking advancements in communication technologies, catalyzing the emergence of mission-critical applications with sub-millisecond latency constraints and microsecond-level timing synchronization requirements. Representative use cases including relay protection systems [14], telesurgery platforms [15], and industrial cyber–physical control systems impose differentiated network performance thresholds [16]. For instance, telemedicine applications mandate end-to-end latency below 20 ms, as exceeding this threshold could critically compromise surgical intervention viability. Similarly, differential protection mechanisms require 200 μs-level latency guarantees to ensure substation fault detection precision, where timing deviations exceeding 50μs may trigger protection misoperations. These stringent requirements underscore the imperative to pioneer low-latency fine-grained network slicing architectures as a cornerstone technology for 6G network infrastructure.

**Figure 1:**
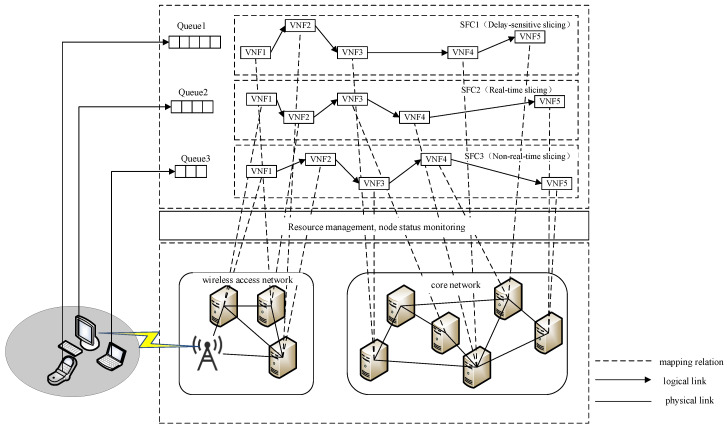
Schematic of fine-grained low-latency network slicing for 6G networks based on GCN.

The system architecture under investigation, as depicted in Figure 1, is formally structured through three principal strata: terminal devices, the radio access network, and the core network infrastructure. The RAN subsystem implements intelligent access technology selection mechanisms to optimize user equipment connectivity, while the core network orchestrates SFC mapping operations. Three distinct network slice categories are provisioned, all operating under latency-bound SLAs (Service-Level Agreements) but with differentiated computing and communication resource allocations: LSSs (latency-sensitive slices), RTSs (real-time slices), and NRTSs (non-real-time slices). Service reliability is strictly contingent upon the slice’s capability to satisfy predefined latency thresholds through resource reservation. Furthermore, application-specific SFCs are dynamically instantiated per slice category, ensuring service execution compliance with heterogeneous requirements. The architecture establishes deterministic E2E communication paths spanning from user terminals through base stations to core network elements, implementing coordinated resource reservation across all network strata.

Within the RAN domain, protocol layer function virtualization is achieved through DU (Distributed Unit) devices leveraging standardized server clusters, establishing a centralized processing pool. To address heterogeneous network slicing requirements, an adaptive VNF (Virtual Network Function) deployment framework is implemented across SFCs, enabling joint optimization of computational resource allocation and infrastructure utilization efficiency. Capitalizing on the inherent caching capabilities of RAN infrastructure, per-SFC packet scheduling mechanisms are provisioned at DU endpoints to refine network resource allocation granularity and maximize system throughput. In the core network stratum, the software-defined virtualization of physical switching/routing devices is realized via commodity server platforms. VNF services within SFCs are instantiated through cloud-native network functions deployed on VMs (Virtual Machines), with cross-VM elastic VNF orchestration enabling multidimensional service provisioning while maintaining optimal resource allocation efficiency across heterogeneous infrastructure.

This section conducts a systematic latency taxonomy analysis, establishing three service classification tiers with corresponding provisioning frameworks. Service instances are classified into three distinct categories: NRTS, RTS, and LSS. The classification criteria are defined through rigorous latency-bound thresholds: NRTSs accommodate applications with relaxed latency constraints exceeding 100 ms; RTSs require bounded latency between 10 ms and 100 ms to maintain service continuity; and LSSs mandate ultra-strict latency guarantees below 10 ms to support mission-critical operations. Each service category is subsequently associated with dedicated network slice configurations and resource reservation mechanisms to ensure service-specific QoS compliance. LSSs have the highest resource allocation level, which allocates dedicated PRBs (Physical Resource Blocks) and computational resources. RTSs have medium resource allocation levels, for which the system reserves a portion of bandwidth in advance. NRTSs have the lowest resource allocation priority level, and we allow resources to be shared by other slices when NRTSs are available.

The infrastructure network can be abstracted as an undirected weighted graph, denoted as $G_{P}=(N_{P},E_{P},C_{P},B_{P})$. In this model, $N_{P}$ represents the set of physical nodes, which consists of two types: wireless access network nodes ($N_{a}$) and core network nodes ($N_{c}$). $E_{P}$ represents the set of physical links, which includes two categories: physical links between wireless access network nodes ($E_{a}$) and physical links between core network nodes ($E_{c}$), each fulfilling distinct network connectivity requirements. $C_{P}$ denotes the set of computational resources of the physical nodes, while $B_{P}$ represents the set of bandwidth resources of the physical links. The network slice request set consists of three types of slices, denoted as *R_NS_*, where *R_NS_* = *R_NRT_∪R_RT_∪R_CT_*. Here, R_NRT_ represents non-real-time slices, R_RT_ represents real-time slices, and R_CT_ represents latency-sensitive slices. The set of slice type is denoted as $M=\left\{1,2,\cdots ,m\right\}$. Each request is represented as *G_R_* = (*N_R_*, *E_R_*, *C_R_*, *B_R_*, *T_R_*). Each slice type consists of a specific set of corresponding VNFs. The VNF composition for slice m is denoted as $N_{I}^{V_{m}}=\left\{N_{1}^{V_{m}},N_{2}^{V_{m}},\cdots ,N_{i}^{V_{m}}\right\}$. In this section, a graph structure is employed to represent the SFC of service requests. Specifically, service requests are abstracted as a DAG $G_{V}=(N_{V},E_{V})$, where $N_{V}$ represents the set of virtual nodes, and $E_{V}$ represents the set of virtual links.

### 2.1. Latency Definition

In the access network, assume that multiple Remote RRUs (Remote Radio Units) are distributed within a region, forming the set $J=\left\{1,2,\cdots ,j\right\}$. To efficiently manage and utilize wireless resources, the total bandwidth B Hz is divided into multiple PRBs, represented by the set $P=\left\{1,2,\cdots ,p\right\}$, where different PRBs are orthogonal to each other. Let $P_{j,l,u^{m}}$ denote the power allocated by *RRU*j on *PRB*l to user $u^{m}$ in slice m, and $h_{j,l,u^{m}}$ represents the corresponding channel gain. It is important to note that a user can connect to more than one RRU, and different PRBs from different RRUs can be allocated to the same user. Therefore, the rate provided to user $u^{m}$ in slice m by *RRU*j on *PRB*l can be expressed as

(1) $$r_{j,l,u^{m}}=b{log}_{2}\left(1+\frac{P_{j,l,u^{m}}h_{j,l,u^{m}}}{\sum_{\forall j^{\prime }\epsilon J,j^{\prime }\neq j}\sum_{\forall m\in M}\sum_{\forall v^{m}\in U^{m},v^{m}\neq u^{m}}P_{j^{\prime },l,v^{m}}h_{j^{\prime },l,v^{m}}+\sigma^{2}}\right),$$

where b represents the bandwidth allocated by RRUj on PRBl to user $u^{m}$ in slice m, and $\sigma^{2}$ denotes the noise power.

For slice m, assume that the arrival of SFC packets follows a dynamic Poisson distribution, where the parameter $\lambda_{m(t)}$ varies over time to describe the changing arrival rate of packets in slice m. Additionally, the packet size is characterized by an exponential distribution, with a mean value of $\bar{P_{m}}$.

The rate $r_{j,l,u^{m}}$ provided by *RRU*j on *PRB*l to user $u^{m}$ in slice m is considered the service rate $R_{m(t)}$ of the link. The average packet processing rate is then given by

(2) $$V_{m(t)}=\frac{R_{m(t)}}{\bar{P_{m}}},$$

Let the queue length of slice m in the SFC at time slot t be $q_{m(t)}$. The queue update equation for the SFC on the DU side is then expressed as

(3) $$q_{m(t+1)}=max\left\{q_{m\left(t\right)}+a_{m\left(t\right)}-d_{m\left(t\right)},0\right\},$$

Here, $a_{m\left(t\right)}=\lambda_{m(t)}\cdot T_{s}$ represents the number of data packets arriving in the time slot. Additionally, $d_{m\left(t\right)}=V_{m(t)}\cdot T_{s}$ denotes the number of data packets processed in the time slot.

Based on Little’s Law, the average number of objects in a system can be derived by calculating the product of the average arrival rate of the objects and their average residence time within the system. Therefore, the queuing delay can be expressed as

(4) $$D_{queue}^{m}=\sum_{m\epsilon M}\frac{q_{m\left(t\right)}}{\lambda_{m(t)}},\;$$

The processing time required by a physical network node n after receiving a VNF data packet $N_{I}^{V}$ is defined as the node processing latency. For slice m, this latency is expressed as

(5) $$D_{proc}^{m}=\sum_{N_{I}^{V_{m}}\in N_{v}}\sum_{N_{n}^{P}\in N_{P}}\delta_{i}^{n}\;\frac{X_{i}^{m}}{R_{n}^{m}},$$

where $\delta_{i}^{n}\in \left\{0,\;1\right\}$ is defined to indicate whether the *i*-th VNF is deployed on server n. Specifically, $\delta_{i}^{n}=1$ if the *i*-th VNF is deployed on server n, and $\delta_{i}^{n}=0$ otherwise. $R_{n}^{m}$ represents the computational processing capability of node *n* in slice m, while $X_{i}^{m}$ denotes the packet size required by the *i*-th VNF $N_{i}^{V}$ in slice m.

The time required to transmit the data packets of VNF $N_{i}^{V}$ between physical network nodes through network links is referred to as the link transmission delay. The transmission delay for slice m is expressed as

(6) $$D_{tran}^{m}=\sum_{N_{I}^{V_{m}}\in N_{v}}\sum_{N_{n}^{P}\in N_{P}}\delta_{i}^{n}\delta_{i}^{n^{\prime }}h_{n,n^{\prime }}\frac{X_{i}^{m}}{R_{n,n^{\prime }}^{m}},$$

where the $\delta_{i}^{n^{\prime }}$ indicates whether the *i*-th VNF is deployed on server $n^{\prime }$; $h_{n,n^{\prime }}$ represents the number of hops between physical nodes n and $n^{\prime }$; and $R_{n,n^{\prime }}^{m}$ denotes the transmission rate between physical nodes n and $n^{\prime }$.

The end-to-end delay for slice *m* is equal to the sum of the node processing delay and the link transmission delay:

(7) $$T^{m}=D_{queue}^{m}+D_{tran}^{m}+D_{proc}^{m}.$$

where $D_{queue}^{m}$ is the queueing delay of slice m in the access network, $D_{proc}^{m}$ is the total node processing delay for slice m in the access and core networks, and $D_{tran}^{m}$ is the total link transmission delay for slice m in the access and core networks.

Therefore, the objective function is

(8) $$min\;T^{m}\;$$

The constraints are

(9) $$\sum_{n\in N_{P}}\delta_{i}^{n}(t)=1,\forall i\in N_{V}$$

(10) $$\sum_{n\in N_{P}}\delta_{i}^{n}(t).C_{i}^{v}\leq C_{n}^{p},\forall n\in N_{p}$$

(11) $$\sum_{i\in N_{V}}(\psi_{ij}^{nm}(t)+\psi_{ij}^{mn}(t))B_{ij}^{v}\leq B_{nm}^{p},\forall n,m\in N_{p},\forall n,m\in L_{P}$$

(12) $$\sum_{i\in N_{V}}\psi_{ij}^{nm}(t)-\sum_{i\in N_{V}}\psi_{ij}^{mn}(t)=\delta_{i}^{n}(t)-\delta_{j}^{n}(t),\forall n,m\in N_{p},\forall nm\in L_{P}$$

(13) $$\delta_{i}^{n}(t)=[0,\;1],\forall i,j\in N_{V},\forall n,m\in N_{p}$$

(14) $$\psi_{ij}^{nm}(t)=[0,\;1],\forall i,j\in N_{V},\forall n,m\in N_{p}$$

(15) $$\theta_{n}(t)=[0,\;1],\forall n\in N_{p}$$

Equation (9) ensures that each VNF on the SFC can only be deployed on one generic server. Equation (10) ensures that the total computational resources required by the VNFs deployed on a given server do not exceed the total computational resources available. Equation (11) guarantees that the sum of bandwidth resources required by all virtual links mapped onto physical links does not exceed the total bandwidth of the physical link. Equation (12) requires that when two adjacent VNFs on the SFC are deployed on servers n and m, respectively, there must be at least one connection path between the physical links nm. Equations (13)–(15) use binary variables to represent the deployment status of VNFs, the mapping of virtual links, and the utilization of generic servers, respectively.

### 2.2. Service Function Chain Construction

Two characteristic relationships are observed between network functions: dependency-based and autonomy-preserving topologies. Upon receiving service requests, the corresponding SFC is initially instantiated through formal graph composition rules [17]. Subsequently, a resource-aware mapping algorithm is implemented to optimally place VNFs along the SFC onto infrastructure nodes, achieving computational load balancing while maintaining service continuity constraints. Virtual machine interconnects are dynamically provisioned based on SFC requirements, establishing end-to-end service paths with customized topological configurations [18]. Each network function is mathematically characterized by two key parameters: $\lambda_{f}\;$, denoting the input/output bandwidth ratio, and $\mu_{f}$, representing the computational resource consumption per 1 Mb/s traffic unit. These parameters remain invariant during data plane processing, strictly adhering to preconfigured network function profiles.

In the process of constructing the SFC, the algorithm first analyzes each service request of users with precision and efficiency, independently constructing a network function dependency graph for each user [19]. Subsequently, the algorithm uses these dependency graphs as input to build an SFC tailored to meet the specific needs of each user.

**Figure 2:**
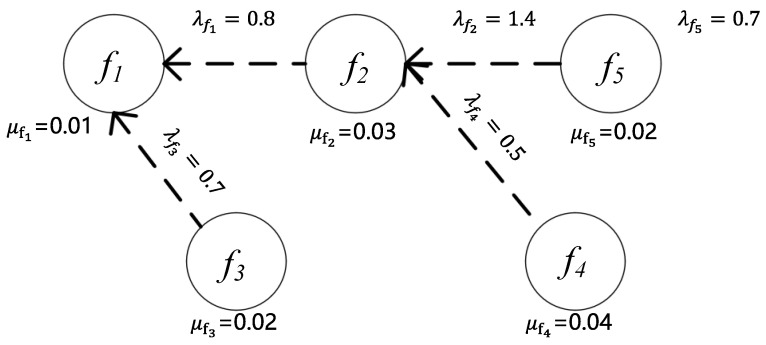
Diagram of network function dependency.

The network function dependency graph used in this section is shown in Figure 2.

The dashed line from $f_{2}$ to $f_{1}$ indicates that in the dependency relationship between $f_{2}$ and $f_{1}$, $f_{2}$ depends on $f_{1}$, meaning that $f_{2}$ must be positioned after $f_{1}$ when constructing the service chain. Here, $\lambda_{f}$ represents the ratio of output to input bandwidth, and $\mu_{f}$ refers to the computational resources required to process a 1 Mb/s flow.

**Figure 3:**
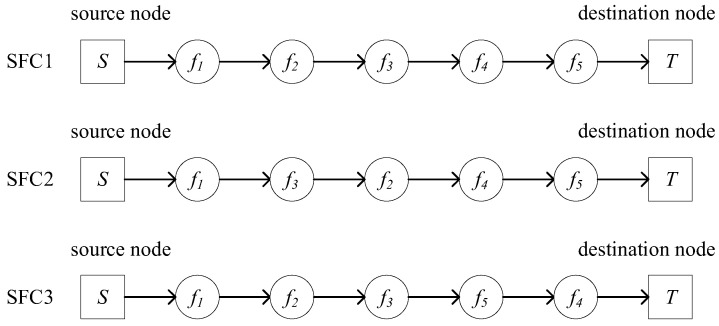
Diagram of the constructed SFCs.

Based on Figure 2, multiple SFC construction schemes can be generated. One such successfully constructed SFC, which satisfies both the network function dependencies and service requirements, is shown in Figure 3. In the service request, key information includes the source node S, destination node T, initial bandwidth requirement $B_{ini}$, and the VNF set.

In a slice network, the set of network functions is represented by F, which includes all the available VNFs on the slice. To clearly describe the dependencies between these functions, a matrix D with |F| rows and |F| columns is used to represent them:

(16) $$D\left(f_{i},f_{j}\right)=\left\{\begin{array}{cc} 1, & f_{i}\;\mathrm{depends}\;\mathrm{on}{\;f}_{j}\\ 0, & \mathrm{other} \end{array}\right.,\;0\leq i,j<\left|F\right|$$

where $C_{total}^{k}$ represents the total computational resources required by the VNF on $C_{K}$, and $C_{K}$ refers to the *K*-th network request. Therefore, the following holds:

(17) $$C_{total}^{k}=\sum_{0\leq i<\left|F^{k}\right|}B_{i-1}^{k}\lambda_{i-1}\mu_{i},$$

where $B_{total}^{k}$ represents the total bandwidth resources required by the VNFs in $C_{K}$. Thus, the relationship can be expressed as

(18) $$B_{total}^{k}=B_{ini}+\sum_{0\leq i<\left|F^{k}\right|}B_{i-1}^{k}\lambda_{i},$$

where $V^{k}$ represents the evaluation value of the SFC constructed for $C_{K}$ in terms of both computational and bandwidth resources:

(19) $$V^{k}=\alpha C_{total}^{k}+\beta B_{total}^{k}.$$

Finally, the SFC corresponding to different evaluation values is selected based on the actual requirements.

The core idea of the SFC construction algorithm based on the depth-first search is as follows: Firstly, the dependencies and constraints between the VNFs within the sliced network are extracted and represented in a tree structure, where each node represents a VNF and the connections between the nodes reflect the dependencies among the VNFs. Secondly, due to the dependencies between the VNFs, they are positioned at different hierarchical levels. The algorithm selects the VNF node at the highest level as the starting point. Then, starting from this node, the algorithm traverses the tree in reverse order to reach the root node. This backtracking process aims to determine the initial path of the SFC, which is the path connecting the source node to the highest-level VNF. Once the initial SFC is determined, the algorithm proceeds to further search and add the associated sibling nodes.

**Figure 4:**
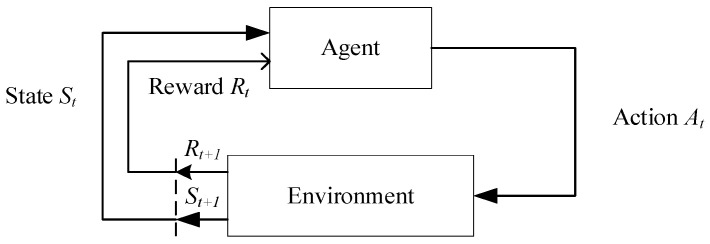
Schematic diagram of the MDP.

The SFC mapping problem has been proven to be NP-hard, meaning that finding the optimal solution is computationally challenging [20]. The objective of the optimization is to minimize latency by integrating the resource and topology information of the entire infrastructure network and the constructed SFCs, forming a comprehensive system state. The SFC mapping problem is inherently a complex and computationally intensive decision-making process, which aligns with the definition of an MDP (Markov Decision Process) [21]. Given that MDPs are effective in describing decision-making problems with state transitions and reward mechanisms, reinforcement learning methods can be employed to solve this problem. The interaction process between the agent and the environment is shown in Figure 4.

The MDP consists of five key components, which can be abstractly represented as (S,A,P,R,γ). S represents the state space, A denotes the action space, P represents the state transition probability, R is the reward value, and γ represents the discount factor. At each time step τ, the DRL (Deep Reinforcement Learning) agent observes the state $S_{\tau }$ and selects an action $a_{\tau }$. After the agent acts, the environment transitions to a new state $S_{\tau +1}$, and the agent receives a reward $r_{\tau }$.

The state $s_{t}$ represents the environment at a specific time t, which can be either discrete or continuous data. The set of all possible states forms the state space S. Actions $a_{t}$ describe the behaviors executed by the agent at a particular moment t, and the collection of all possible actions constitutes the action space A. The policy $\pi \left(\left.a_{t}\right|s_{t}\right)$ is a function that determines the next action $a_{t}$ based on the current state $s_{t}$. The state transition probability $p\left(\left.s_{t+1}\right|s_{t},a_{t}\right)$ indicates the likelihood that the environment transitions to state $s_{t+1}$ after the agent takes action $a_{t}$ in state $s_{t}$ at time t. The reward $R_{t}$ is the feedback the environment provides after the agent performs action $a_{t}$ in state $s_{t}$. This reward is not only related to the current action but also closely tied to the resulting state $s_{t+1}$ at the next time step.

To evaluate the performance of the policy $\pi \left(\left.a_{t}\right|s_{t}\right)$, the agent aims to maximize its long-term expected return by executing a sequence of actions. To achieve this objective, the concept of the state–action value function is introduced, which quantifies the long-term reward for taking a specific action in a given state. By properly defining and optimizing this function, the agent can make more informed decisions and thereby achieve higher returns. The state–action value function $Q^{\pi }\left(s,a\right)$ represents the expected return when the agent, starting from state $s_{t}$, takes action under the policy $\pi \left(\left.a_{t}\right|s_{t}\right)a_{t}$. Its mathematical expression is given as follows: $Q^{\pi }\left(s,a\right)=E_{\pi }(G_{t}|s_{t}=s,a_{t}=a)$. Here, $G_{t}=R_{t+1}+\gamma R_{t+2}+\gamma^{2}R_{t+3}+\cdots$, and γ is the discount factor for future rewards, with γ∈[0,1). The term $G_{t}$ represents the discounted total return. The above value function can be decomposed using the Bellman equation as follows: $Q^{\pi }\left(s_{t},a_{t}\right)=R_{t}+\sum_{s_{t+1}\in S}P_{s_{t}s_{t+1}}^{a_{t}}\sum_{a_{t+1}\in A}\pi \left(\left.a_{t+1}\right|s_{t+1}\right)Q^{\pi }\left(s_{t+1},a_{t+1}\right)$.

### 2.3. Algorithmic Analysis

The following section illustrates the system states, actions, and reward functions in the deployment of SFCs for low-latency services within fine-grained network slicing in 6G networks. A GCN is a neural network that can directly act on domain graph structural data and can fully utilize its structural information. In the realization of network slicing technology, network topology is crucial, and a GCN can effectively handle complex network topology structure information to better realize the construction and deployment of network slices [22]. It can rely on its powerful capabilities to help in multiple stages of the network slicing orchestration and deployment process, such as slicing design [23], slicing deployment [24], and slicing performance and alarm monitoring [25]. Based on this, a GCN-based algorithm for 6G network low-latency service fine-grained network slicing is proposed.

*A.*  *State Space*

The state space provides a comprehensive description of various resources across the network and the current processing state of each VNF, which is defined as $S(t)=\{C\left(t\right),M\left(t\right),B\left(t\right),\delta (t)\}$. At time t, C(t) represents the remaining computational resource vector across all nodes; M(t) shows the remaining storage resources at the nodes; and B(t) is the vector of the remaining bandwidth on the links between nodes. If two nodes are not directly connected by a link, the corresponding value of $B_{n_{x}n_{y}}^{res}$ will remain 0; δ(t) is a vector composed of binary variables that represent the mapping states of each node, describing the mapping states of the VNFs within the entire SFC.

*B.*  *Action Space*

When selecting the next node for mapping, the set of selectable nodes consists of all the neighbor nodes directly connected to the current node via physical links. The action space is determined and constructed by all the VNFs currently mapped on the nodes. The vector A(t) represents the action space at time *t* and is defined as $A(t)=[A_{n_{1}}(t),A_{n_{2}}(t),\cdots ,A_{n_{\left|N\right|}}(t)]$, where $A_{n_{x}}(t)$ denotes the set of possible next-hop actions for all the VNFs already mapped at node $n_{x}$.

*C.*  *Reward Function Definition*

The agent continuously receives rewards $r_{\tau }$ from the external environment to enhance its performance and train its neural network, rather than following predefined labels. Actions that result in lower latency are considered better, and the environment provides a higher positive reward for such actions. In contrast, infeasible actions (i.e., those that violate at least one constraint) are regarded as incorrect, and the environment returns a reward of 0. Thus, the reward function guides the algorithm toward optimization in the direction of minimizing latency. Based on the previous discussion, the reward $r_{\tau }$ for a given action $a_{\tau }$ at time *τ* is defined as follows:

(20) $$r_{\tau }=\left\{\begin{array}{c} \frac{1}{T},\;\;\;\;\;\;\;\;\mathrm{Feasible}\;\mathrm{actions}\\ 0,\;\;\;\;\;\;\;\mathrm{Infeasible}\;\mathrm{actions} \end{array}\right.$$

The strategy and target value networks of the Double DQN are improved to integrate the Double DQN with GCNs. The features of these nodes are organized into an N×D matrix X. The connections between the nodes are represented by an N×N matrix A, referred to as the adjacency matrix. This adjacency matrix A and the feature matrix X together form the input data for this proposed model, which will be used in subsequent analyses and computation.

**Figure 5:**
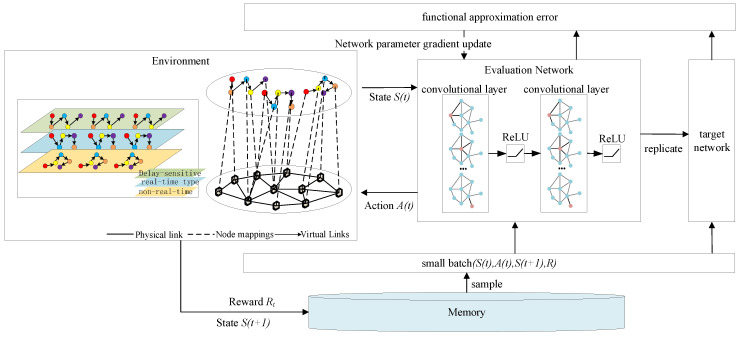
Architecture diagram of the GCN-based low-latency service fine-grained network slicing algorithm.

In this section, modifications are made to the strategy network and the target value network in accordance with the characteristics of the GCN. We modified the layer structure of the neural network to use two convolutional layers, two activation function layers, and ReLu for the activation function. The propagation formula between the layers of the GCN is as follows: $H^{\left(l+1\right)}=\sigma ({\tilde{D}}^{-\frac{1}{2}}\tilde{A}{\tilde{D}}^{-\frac{1}{2}}H^{\left(l\right)}W^{\left(l\right)})$, $\tilde{A}=A+I$, I is the identity matrix, and $\tilde{D}$ is the degree matrix of $\tilde{A}$. H represents the features at each layer, W denotes the weight matrix for the connected edges, and σ is the nonlinear activation function. The architecture diagram of the GCN-based low-latency service fine-grained network slicing algorithm is shown below (Figure 5):

The Double DQN further optimizes the Q-value estimation by decoupling the action selection process from the Q-value computation process, addressing the “overestimation” issue in the DQN. Meanwhile, the GCN, by introducing the adjacency matrix, enhances the ability to extract features from the graph. The specific execution steps of the GCN-based low-latency service fine-grained slicing algorithm in a 6G network are outlined as follows:

Step 1: Initialize the capacity of the experience pool and set the initial weights of the Q-value network and the target-value network.

Step 2: Map the service function chain constructed for the current service according to Algorithm 1 during each training process, and repeat step 3 through step 5 during the network training process until the whole network reaches a converged state.

Step 3: According to the current network state S(t), based on the predefined ε-strategy, select and execute the action A(t) in the action space and then observe the state change of the network to enter a new state S(t+1).

Step 4: Obtain the reward value $R_{t}$ from the executed action and update the Q-value network parameters. Then, update the weights of the target value network.

Step 5: Deposit the samples $\left(S(t),A(t),S(t+1),R_{t}\right)$ into the experience pool from which samples are subsequently randomly drawn for subsequent model training and updating.
**Algorithm 1:** A Deep-First Search-Based Algorithm for Constructing Low-Latency Fine-Grained Network Slicing in 6G NetworksInput: *F*, *D*
Output: SFC construction scheme and its evaluation value
$V^{k}$
1: Transform the dependency *D* into a tree structure2: for
k←0
 to |c|
3:   From the node where $q_{m}^{k,h}$ is located to find the parent node, grandfather node and root node, and put these nodes into the set $q_{node}$;4:   $q_{init}=q_{node}\cap F^{k}$.5:   Assign the remaining elements in $F^{k}$ to set $q_{rem}$;6:   $Deepfc.copy(q_{init})$;7:    for i←0
 to Temp.size()
8:    Temp.copy(Deepfc);9:    
Deepfc.clear()
10:     for
j←0
 to Temp.size()
11:      for
m←0
 to Temp[j].size()
12:       t=Temp build a SFC according to the residual node rule.13:       Deepfc.copy(Temp[j]);14:       Delete the node added by Temp[j].15:      end for16:     end for17:     end for18: Equations (17) and (18) were used to compute the required computing resources and bandwidth resources.19: Equation (19) was used to compute evaluation value $V^{k}$.20: end for

## 3. Results

The time and space complexity of the proposed algorithm can be calculated in two parts: the time complexity of Algorithm 1 is $O=({\left|F\right|}^{3})$, and the space complexity is O=(|F|). Algorithm 2 has a time complexity $O=(T\cdot ({\left|F\right|}^{3}+|A|))$ and a space complexity $O=(C\cdot (\left|F\right|+|E|))$, where |A| is the size of the action space, T is the number of training rounds, |E| is the number of dependent edges, and C is the capacity of the playback experience pool, so the total time complexity of the algorithm is $O=({\left|F\right|}^{3}+T\cdot ({\left|F\right|}^{3}+|A|))$ and the total space complexity is $O=(\left|F\right|+C\cdot (|F|+|E|))$. The algorithm satisfies polynomial time in theoretical complexity and is suitable for dealing with medium-sized and below network problems.
**Algorithm 2:** A GCN-Based Algorithm for Constructing Low-Latency Fine-Grained Network Slicing in 6G Networks1: Randomly reply memory to capacity *D*.2: Initialize the Q-network Q with random weights θ.3: Initialize the target Q-network $\hat{\theta }$ with random weights $\hat{\theta }=\theta$.4: For episode t=1 to T do5:     According to Algorithm 1, a dedicated network SFC with source node and end node is generated for the current service.6:    With probability ε select a random action A(t)$,\;\mathrm{otherwise}\;\mathrm{select}\;A\left(t\right)={argmax}_{A\left(t\right)\in a}Q(S\left(t\right),A\left(t\right);\theta )$.7:      Perform action $A\left(t\right)$ and get the reward $R_{t}$, observer the next state S(t+1).8:      Store transition $\left(S(t),A(t),S(t+1),R_{t}\right)$ in the replay memory.9:      Sample random minibatch of transitions $\left(S(j),A(j),S(j+1),R_{j}\right)$ from the replay memory.10:     $y_{j}=R_{j}+\gamma Q(S\left(j+1\right),{\;argmax}_{A\left(j+1\right)\in a}\hat{Q}\left(S\left(j+1\right),A\left(j\right);\theta \right);\hat{\theta })$.11:     Perform a gradient step on ${\left(y_{j}-Q\left(S\left(j\right),A\left(j\right);\theta \right)\right)}^{2}$ with respect to the network parameter θ.12:     Every step reset $\hat{\theta }=\theta$.13: end for

The underlying random physical network topology is constructed using the NetworkX library. NetworkX is a package for Python 3.9; in this paper, we set the network topology as a Barabasi–Albert network by NetworkX, and the weight change of the edges is set as a sinusoidal function fluctuation mode. Other simulation parameters are set as shown in Table 1.

The following section compares the proposed GCN-based low-latency fine-grained network slicing algorithm in 6G networks with the Double DQN algorithm.

**Figure 6:**
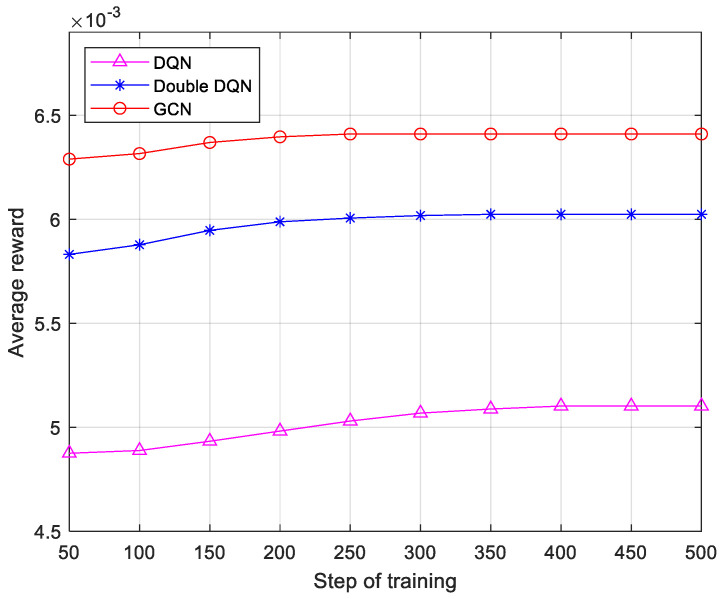
Convergence speed graph.

As shown in Figure 6, the reward value in this study is defined as the inverse of transmission time. Sixth-generation networks need to support highly dynamic, low-latency services. Algorithms must quickly adjust their strategies in the rapidly changing network environment. The convergence speed directly reflects the time required for the algorithm to stabilize the optimal state from the initial state and is a key indicator of the algorithm’s real-time and adaptive ability. The simulation results show that the DQN algorithm converges at 400 training steps, the Double DQN algorithm converges at 300 training steps, and the proposed algorithm achieves convergence in just 250 steps. In addition, the algorithm proposed in this paper improves the reward by 25.74% and 8.50% over the DQN and Double DQN, respectively. This means that the algorithm proposed in this paper is more suitable for resource-sensitive communication scenarios compared to the DQN and Double DQN.

**Figure 7:**
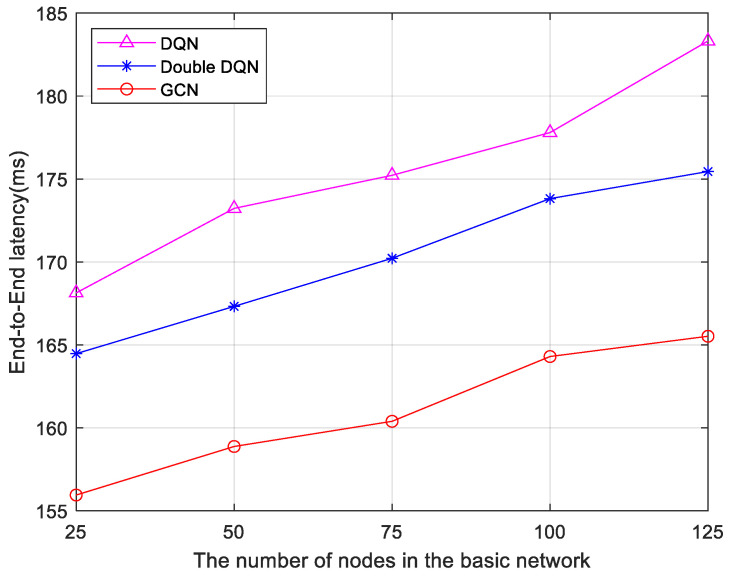
Comparison of latency with various numbers of basic network nodes.

Furthermore, three algorithms are compared based on delay data under five different infrastructure network node configurations. The results are shown in Figure 7. As the number of infrastructure network nodes increases, the end-to-end delay increases. However, the end-to-end delay of the 6G network low-latency service fine-grained slicing algorithm based on the GCN remains lower than both the Double DQN algorithm and the DQN algorithm. This is because the algorithm used in this paper itself takes into account the network topology, which has a deeper message propagation depth and has higher network scalability, so the increase in nodes has less impact on latency. This indicates that the GCN-based algorithm possesses greater stability as the number of nodes in the network topology changes.

**Figure 8:**
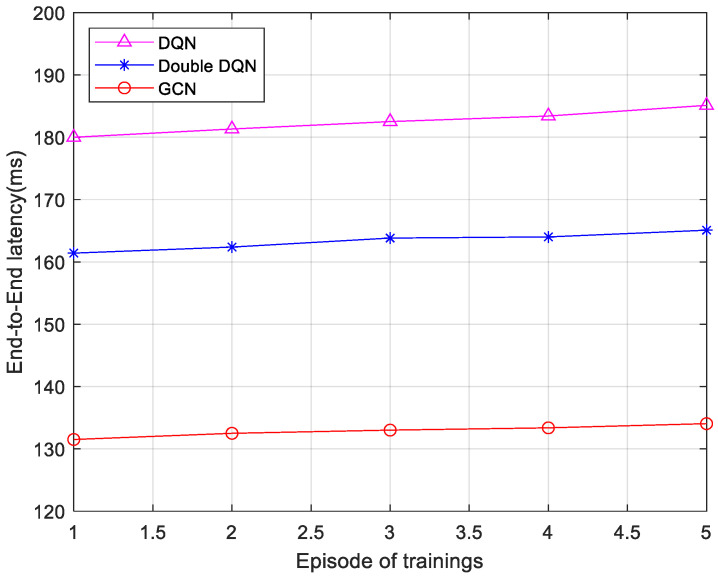
Comparison of end-to-end delay for service size 25.

**Figure 9:**
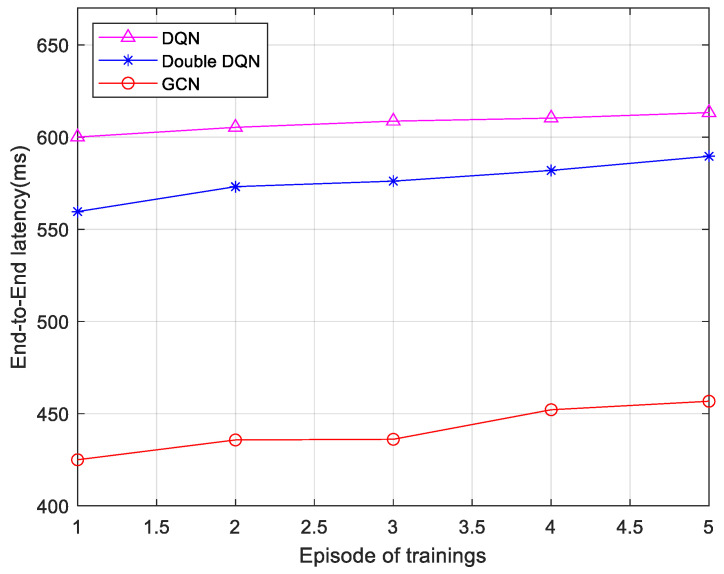
Comparison of end-to-end delay for service size 75.

Figure 8 and Figure 9 present comparisons of the end-to-end delay for service execution under three different algorithms, with service sizes of 25 and 75, respectively. The service size plays a crucial role in resource allocation strategies. A larger service size typically necessitates more computational and bandwidth resources, which place greater demands on the dynamism and coordination of resource allocation strategies. For each scenario, five sets of average end-to-end latency data were collected from five rounds of training, with each round consisting of 200 time steps. As observed in the figures, the end-to-end latency of all three algorithms increases as the size of the service data packet grows. The GCN-based algorithm reduces the latency by 26.92% over the DQN and 18.77% over the Double DQN when the service size is 25. When the service size is 75, this algorithm reduces the latency by 28.82% and 22.41% compared to the DQN and Double DQN, respectively. The reason for this is that the algorithm proposed in this section leverages the spectral properties of the graph and operates on the Laplace matrix to update node features, which enhances computational capability. Therefore, the end-to-end latency of the 6G network low-latency service fine-grained slicing algorithm based on the GCN is lower than that of the Double DQN algorithm and the DQN algorithm.

## 4. Conclusions

In this paper, we propose a GCN-based low-latency service fine-grained network slicing algorithm for 6G networks. First, a finer-grained latency-type slicing approach is introduced, which comprehensively considers both the computing and communication resources of the RAN and core network in the process of calculating end-to-end latency. The simulation results show that this algorithm fully leverages network topology information and incorporates local and neighboring characteristics, significantly reducing end-to-end latency. This indicates that compared to the DQN and Double DQN algorithms, the algorithm proposed in this paper enhances the ability to model the network topology, which significantly improves the effective utilization of resources by capturing the local and global resource dependencies.

Additionally, a deployment method for the constructed network slices is presented. During the deployment process, a novel approach is presented to represent the diversified and complex 6G service requests using DAGs. The DAG-based service request representation enables accurate modeling of complex 6G service dependencies. By abstracting service requests as DAGs, the algorithm can capture intricate VNF interdependencies and topological constraints, thus facilitating efficient SFC construction. A depth-first search-based SFC generation mechanism further ensures compliance with hierarchical functional dependencies. Furthermore, the graph data feature extraction capabilities of GCN are utilized to address the challenges posed by the DAG, thereby enabling effective deployment of the SFC.

In summary, this work advances 6G network slicing techniques by addressing the limitations of coarse-grained resource allocation and linear SFC models. Our future research will explore real-world testbed implementations and extend the framework to support more complex 6G network slicing scenarios.

## Figures and Tables

**Table 1:** Simulation parameter list.

Network Parameters	Values
Total storage resources of physical nodes	Uniform distribution with mean 100 and variance 30
Total computing resources of physical nodes	Uniform distribution with mean 150 and variance 40
Base station wireless channel bandwidth (MHz)	20
Noise power spectral density (dBm/Hz)	−174
Number of VNF types	5
Length of SFC	[5, 10]
Total number of deployed SFCs	[50, 80]
Number of VNFs in each SFC	5

## Data Availability

The original contributions presented in this study are included in the article. Further inquiries can be directed to the corresponding author.
